# Postnatal care utilisation among women in rural Ghana: analysis of 2014 Ghana demographic and health survey

**DOI:** 10.1186/s12884-020-03497-4

**Published:** 2021-01-07

**Authors:** Francis Appiah, Tarif Salihu, Justice Ofosu Darko Fenteng, Andrews Ohene Darteh, Patience Kannor, Patience Ansomah Ayerakwah, Edward Kwabena Ameyaw

**Affiliations:** 1 Department of Population and Health, University of Cape Coast, Cape Coast, Ghana; 2 Berekum College of Education, Berekum, Bono Region Ghana; 3 Department of Community Medicine, School of Medical Sciences, University of Cape Coast, Cape Coast, Ghana; 4 Department of Optometry, University of Cape Coast, Cape Coast, Ghana; 5 School of Public Health, Faculty of Health, University of Technology Sydney, Sydney, Australia

**Keywords:** Prevalence, Correlates, Postnatal care, Rural women, Reproductive health, Public health, Ghana

## Abstract

**Background:**

Maternal mortality is high in Ghana, averaging 310 maternal deaths per 100,000 live births in 2017. This is partly due to inadequate postnatal care especially among rural communities. Ghana can avert the high maternal deaths if women meet the World Health Organisation’s recommended early postnatal care check-up. Despite the association between geographical location and postnatal care utilisation, no study has been done on determinants of postnatal care among rural residents in Ghana. Therefore, this study determined the prevalence and correlates of postnatal care utilization among women in rural Ghana.

**Methods:**

The study utilised women’s file of the 2014 Ghana Demographic and Health Survey (GDHS)*.* Following descriptive computation of the prevalence, binary logistic regression was fitted to assess correlates of postnatal care at 95% confidence interval. The results were presented in adjusted odds ratio (AOR). Any AOR less than 1 was interpreted as reduced likelihood of PNC attendance whilst AOR above 1 depicted otherwise. All analyses were done using Stata version 14.0.

**Results:**

The study revealed that 74% of the rural women had postnatal care. At the inferential level, women residing in Savanna zone had higher odds of postnatal care compared to those in the Coastal zone [AOR = 1.80, CI = 1.023–3.159], just as among the Guan women as compared to the Akan [AOR = 7.15, CI = 1.602–31.935]. Women who were working were more probable to utilise postnatal care compared to those not working [AOR = 1.45, CI = 1.015–2.060]. Those who considered distance as unproblematic were more likely to utilise postnatal care compared to those who considered distance as problematic [AOR = 1.63, CI = 1.239–2.145].

**Conclusions:**

The study showed that ethnicity, ecological zone, occupation and distance to health facility predict postnatal care utilisation among rural residents of Ghana. The study points to the need for government to increase maternal healthcare facilities in rural settings in order to reduce the distance covered by women in seeking postnatal care.

**Supplementary Information:**

The online version contains supplementary material available at 10.1186/s12884-020-03497-4.

## Background

Globally, 61% of the 585,000 annual maternal deaths occur within the postnatal stage whereas more than half of these transpire within the first day of childbirth [1]. The situation is worrying in sub-Saharan Africa (SSA) where 66% of the global maternal deaths occurred in 2017 [2–4]. Inaccessibility and poor postnatal care (PNC) utilisation account for 99% of maternal mortality in low-middle-income countries (LMICs) including SSA, where majority of childbirths occur at home [2–5].

Similarly, in the Ghanaian context, maternal mortality remains high averaging 310 maternal deaths per 100,000 live births [6] partly due to inadequate postnatal care especially among rural communities [7, 8]. Therefore, Ghana can avert the higher maternal morbidity and associated deaths if women are able to meet the WHO recommended early PNC check-up [9–11] which could also facilitate the realization of the Sustainable Development Goal (SDG) 3 [12]. In LMICs, including Ghana, puerperal infections are sometimes undiagnosed due to inadequate PNC follow-up and most postnatal infections occur after being discharged from hospital, which is mostly 24 h after birth [2].

Conventionally, postnatal stage begins immediately after childbirth until 6 weeks (42 days) after birth [13]. The WHO has advised that women should receive at least three postnatal care visits in addition to the first visit which is expected to take place within 24 h of birth. The second visit should fall on day 3, third visit between day 7 and 14, and the last visit before the end of the 6th week [11]. PNC is critical as it helps health professionals to provide comprehensive reproductive health service for women and their babies [1, 2, 14]. During PNC, health professionals are able to evaluate and verify bleeding, examine the breast, control anemia, encourage nutrition and insecticide bed nets, and also educate women on early and exclusive breastfeeding and umbilical cord care [1, 2, 14]. Additionally, through PNC, babies receive services such as birth registration, screening and infection treatment, postnatal growth monitoring and routine immunization services [1, 2, 14].

In spite of these benefits, most women in rural communities are unable to attend PNC [2, 15, 16]. The 2014 Ghana Demographic and Health Survey (GDHS) indicated that 74% of mothers living in rural areas were least probable to receive early postnatal check-up relative to other subgroups. Women in rural areas of Ghana travel 4 km more than urban women to reach a hospital [17]. The same study indicated that a kilometre increase in distance significantly reduces maternal healthcare utilisation [17]. Additionally, it is known that the distribution of health facilities is skewed towards urban centres in Ghana [18]. This presupposes that women in rural communities may be challenged in accessing PNC compared to women in urban locations of the country.

Despite the association between geographical location and PNC utilisation, no national study has been done on determinants of PNC among rural residents in Ghana. Although Adu and colleagues [19] investigated the effects of individual and community-level factors on maternal health outcomes in Ghana and found that rural dwellers were less likely to give birth in health facilities and have PNC compared to urban dwellers, residence was an explanatory variable. Sakeah et al. [20] also explored the role of community-based health planning and services (CHPS) in influencing PNC visits in rural Builsa and the West Mamprusi districts (both in one of the 16 regions) of Ghana and found that women who attended antenatal clinics at least four times and women who had partners with secondary education were associated with at least three PNC visits.

Health facilities and health personnel are concentrated in urban Ghana [18] and as such national level study is required to understand the factors affecting PNC of the rural populace in Ghana. It is against this background that this study seeks to determine the prevalence and correlates of PNC utilization among women in rural Ghana. In the light of the global commitment towards ensuring quality life for persons of all ages [12], this study is of critical public health importance to Ghana. It could guide the Health Promotion and Education Unit and Reproductive and Child Health Department of the Ghana Health Service in preparing and planning for maternal and child health promotion programs that target utilization of maternal health services in rural Ghana.

## Methods

### Data source

The study utilised women’s file of the 2014 GDHS*.* The 2014 GDHS, which is the current and sixth edition of the surveys, captures information on prevention and treatment of malaria for children under five, women’s reproductive performance, family planning, maternal and child health and other information relevant for maternal and child health policies. The implementing partners of the survey include the Ghana Statistical Service (GSS), the Ghana Health Service (GHS), and the National Public Health Reference Laboratory (NPHRL) of the GHS with technical aid from the Inner-City Fund (ICF) International. The survey adopted the Demographic and Health Survey (DHS) standardised questionnaire which is developed by the Measure DHS programme [16].

The 2014 GDHS used an updated sampling frame which was developed by the Ghana Statistical Service for the 2010 Population and Housing Census. This sampling frame do not include nomadic and institutional populations such as persons in hotels, barracks, and prisons. The survey followed a stratified sampling procedure in order to capture specific indicators at the national level whilst taking into account the rural and urban locations [16].

Firstly, sample points, referred to as clusters constituting enumeration areas (EAs) outlined for the 2010 PHC were selected. This resulted to 427 clusters (i.e. 216 and 211 urban and rural clusters respectively). Secondly, a systematic sampling technique was applied to select households and thereafter, a household listing was undertaken in all the selected EAs. Finally, households to be included in the survey were randomly selected from the list. This led to the selection of approximately 30 households from each cluster. In all, 9656 eligible women (comprising 4753 and 4903 women from urban and rural locations respectively) were identified for the survey. However, a total of 9396 women, consisting of 4602 from urban and 4794 from rural settings were interviewed, leading to 97% response rate. However, the current study was restricted to 1442 rural women with complete information on PNC utilisation and the selected explanatory variables. The study was restricted to rural residents because the 2014 GDHS revealed that the proportion of rural residents (21%) who do not obtain PNC are three times more than urban residents who do not obtain PNC (7%) [16]. Additionally, health facilities and health personnel are concentrated in urban Ghana [18]. Further information about the sampling procedure, pre-testing and field activities are available in the 2014 GDHS report [16].

### Definition of variables

#### Outcome variable

The outcome variable for this study was “Postnatal Care (PNC)”. According to WHO, postnatal stage starts immediately after childbirth and goes into 6 weeks (42 days) after childbirth [13]. Therefore, in the DHS Women’s Questionnaire, all women who had a birth in the 5 years preceding the survey were asked whether a health care provider checked them after giving birth or within 2 months after birth accompanied by ‘Yes’, ‘No’ and ‘Don’t Know’. However, for precision in responses, ‘don’t know’ responses were excluded from the analysis. ‘No’ was coded as ‘0’ signifying those who did not receive postnatal check-up and ‘Yes’ as ‘1’, thus those who had postnatal check-up. PNC plays a key role in maternal health by giving women access to varied reproductive health services [1, 2, 14].

#### Independent variables

Sixteen independent variables were selected. These are age, marital status, ecological zone, education, wealth status, religion, ethnicity, occupation, total children ever born, partner’s education, frequency of reading newspaper/magazine, frequency of listening to radio, frequency of watching television, health decision making, holds a valid national health insurance scheme (NHIS) card and getting medical help for self: distance is a problem. For clarity of presentation, education was recoded into no education = 1, primary = 2 and secondary or higher = 3; wealth status was recoded into poor = 1, middle = 2 and rich = 3; region of residence was recoded into the three ecological zones of the country, consisting of Coastal = 1, Middle = 2 and Savanna = 3. Occupation was recoded into not working = 1 and working = 2; religion was recoded into Christian = 1, Islam = 2, Traditionalist = 3 and No religion = 4; total children ever born was recoded into one birth = 1, two births = 2, three births = 3 and four or more births = 4 guided by the current total fertility rate of the country [16]. Partner’s education was recoded into no education = 1, primary = 2 and secondary or above = 3; and finally health decision making capacity was recoded into alone = 1 and not alone = 2. These variables were selected because of their theoretical importance and practical significance to maternal healthcare utilisation [21, 22]. Frequency of reading newspaper/magazine, listening to radio and watching television were included in the analysis because they have been found as significant predictors of antenatal care utilisation and skilled birth attendance [23, 24].

#### Analytical procedure

We first computed the distribution of PNC attendance among women aged 15–49 in rural Ghana. This was followed by a bivariate analysis of socio-demographics and PNC attendance among rural women in Ghana with their respective chi-square of independence test. Since our outcome variable ‘PNC utilisation’ was binary, the binary logistic regression was considered appropriate for the study. This estimation technique was used because it gives room for predictions of outcome variables that are dichotomous in nature. The binary logistic regression was fitted to assess correlates of PNC at 95% confidence interval. Our results was presented in adjusted odds ratio (AOR) and any AOR less than 1 was interpreted as reduced likelihood of PNC attendance whilst AOR above 1 depicts an increased likelihood of PNC utilisation. The weighting factor (v005/100000) inherent in the dataset was applied to cater for the survey sampling errors whilst the ‘linktest’ command and goodness-of-fit were applied to assess the fitness of our model (see Additional files 1 and 2: Appendix 1 and 2 for details). Variance inflation factor (VIF) test for multicollinearity was conducted and the results indicated no evidence of multicollinearity among independent variables (see Additional files 3: Appendix 3). All analyses were done using Stata version 14.0.

#### Ethical considerations

Since the authors of this manuscript did not participate in the actual data gathering processes, we sought no ethical clearance. However, we sought permission to use the data set from Measure DHS. Meanwhile, Measure DHS reported that ethical clearance was obtained from the Institutional Review Board of ICF International and Ethical Review Committee of Ghana Health Service [16]. Also, they ensured that every information that could reveal respondents’ identities were excluded from the dataset before they released the data to the public domain. The data set is freely available to the public at www.measuredhs.org.

## Results

### Descriptive results of the study

**Fig. 1:**
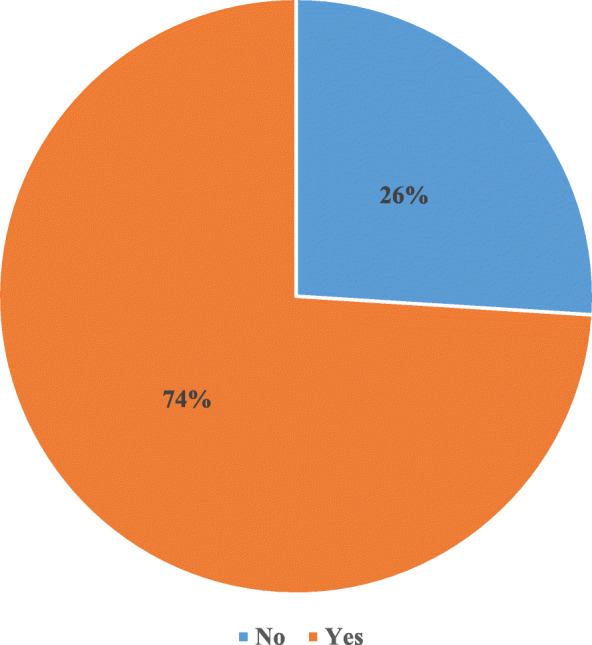
Distribution of PNC attendance among women aged 15–49 in rural Ghana

Figure 1 displays distribution of PNC utilisation among women aged 15–49 in rural Ghana. It was found that majority of them (74%) attended PNC, with 26% professing otherwise.

Table 1 presents the results of socio-demographic characteristics and PNC attendance among women aged 15–49 in rural Ghana. Across the age categories, 83% of those aged 45–49 attended PNC and 77% of married women did the same. It was noted that 87% women in the Savanna zone and 72% of those with secondary/higher education utilised PNC. The analysis revealed that wealthier women (81%) and Muslims (86%) utilised PNC.

**Table 1:** Socio-demographics and PNC attendance among women in rural Ghana (*n* = 1442)

Socio-demographics	Weighted (N)	Weighted (%)	PNC attendance		X^2^(*p*-value)
			No (%)	Yes (%)	
*Age*					4.676 (0.586)
15–19	35	2	27	73	
20–24	231	16	24	76	
25–29	402	28	23	77	
30–34	296	21	27	73	
35–39	300	21	24	76	
40–44	134	9	29	71	
45–49	44	3	17	83	
*Marital status*					13.700(*p* < 0.001)
Married	1100	76	23	77	
Cohabiting	342	24	33	67	
*Ecological zones*					148.524(*p* < 0.001)
Coastal	378	26	19	81	
Middle	601	42	43	57	
Savanna	463	32	13	87	
*Education*					5.580 (0.061)
No education	555	38	23	77	
Primary	284	20	23	77	
Secondary+	603	42	28	72	
*Wealth status*					15.942(*p* < 0.001)
Poor	958	66	23	77	
Middle	299	21	35	65	
Rich	185	13	19	81	
*Religion*					21.595(*p* < 0.001)
Christian	1025	71	27	73	
Islam	301	21	14	86	
Traditionalist	60	4	26	74	
No Religion	56	4	28	72	
*Ethnicity*					77.322(*p* < 0.001)
Akan	541	38	36	64	
Ga/Dangme	55	4	45	55	
Ewe	194	14	29	71	
Guan	33	2	5	95	
Mole-Dagbani	350	24	17	83	
Grusi	51	4	9	91	
Gurma	180	12	25	75	
Mande	19	1	10	90	
Other	19	1	19	81	
*Occupation*					5.332(*p* < 0.05)
Not working	229	16	31	69	
Working	1213	84	24	76	
*Total children ever born*					4.195 (0.241)
One birth	233	16	27	73	
Two births	256	18	20	80	
Three births	263	18	25	75	
Four or more births	690	48	26	74	
*Partner’s education*					15.881(*p* < 0.001)
No formal education	442	31	19	81	
Primary	207	14	26	74	
Secondary+	793	55	29	71	
*Frequency of reading newspaper/magazine*					1.770 (0.413)
Not at all	1338	93	25	75	
Less than once a week	56	4	17	83	
At least once a week	48	3	29	71	
*Frequency of listening to radio*					5.233(*p* = 0.073)
Not at all	277	19	29	71	
Less than once a week	488	34	22	78	
At least once a week	677	47	24	76	
*Frequency of watching television*					3.924 (0.141)
Not at all	641	44	26	74	
Less than once a week	362	25	20	80	
At least once a week	439	31	25	75	
*Health decision making*					10.593(*p* < 0.01)
Alone	310	22	32	68	
Not alone	1131	78	23	77	
*Holds a valid NHIS card*					5.565(*p* < 0.05)
No	191	13	18	82	
Yes	1251	87	26	74	
*Getting medical help for self: Distance is a problem*					8.630(*p* < 0.05)
Big problem	502	35	29	71	
Not a big problem	940	65	22	78	

Source: GDHS 2014

With respect to ethnicity, PNC utilisation was highest among the Guan (95%). PNC utilisation stood at 76% among women who were working whilst 80% of those who had given birth to two children had PNC. Most women whose partners had no formal education utilised PNC (81%). Eight out of ten of the women who read newspaper/magazine less than once a week (83%) utilised PNC. It was found that majority of those that listened to radio less than once a week utilised PNC (78%), just as majority of those who watched television at least once a week (80%), as indicated in Table 1.

PNC utilisation was phenomenal among women who did not take health decisions alone (77%) and those who did not hold a valid NHIS (82%). It was found that 78% of women who perceived distance as unproblematic utilised PNC. With the exception of age, education, total number of children ever born, frequency of reading newspaper, listening to radio and watching television, the other socio-demographics had statistically significant association with the outcome variable as shown in Table 1.

### Correlates of PNC utilisation among rural women aged 15–49 in Ghana

Table 2 depicts correlates of PNC utilisation among women aged 15–49 in rural Ghana. The probability to utilise PNC was higher among women residing in Savanna compared to those in the Coastal zone [AOR = 1.80, CI = 1.023–3.159]. The Guan women were more likely to utilise PNC than the Akan [AOR = 7.15, CI = 1.602–31.935]. More so, women who were working were more probable to utilise PNC as compared to those not working [AOR = 1.45, CI = 1.015–2.060]. Similarly, women who considered distance as unproblematic were more likely to utilise PNC as compared to those who perceived it as problematic [AOR = 1.63, CI = 1.239–2.145]. Finally, from the two model specification diagnoses (see Additional files 1 and 2: Appendix 1 and 2), the final model was well-specified.

**Table 2:** *Correlates of PNC utilisation among rural women aged 15–49 in Ghana*

Socio-demographics	AOR	95% CI
*Marital Status*		
Married	Ref	1,1
Cohabiting	0.96	[0.694–1.329]
*Ecological Zones*		
Coastal	Ref	1,1
Middle	0.28***	[0.190–0.414]
Savanna	1.80*	[1.023–3.159]
*Wealth status*		
Poor	Ref	1,1
Middle	0.85	[0.588–1.222]
Rich	1.53	[0.856–2.749]
*Religion*		
Christian	Ref	1,1
Islam	1.19	[0.780–1.821]
Traditionalist	0.67	[0.367–1.231]
No religion	0.82	[0.437–1.543]
*Ethnicity*		
Akan	Ref	1,1
Ga/Dangme	0.82	‘[0.412–1.643]
Ewe	2.43***	[1.566–3.765]
Guan	7.15***	[1.602–31.935]
Mole-Dagbani	1.24	[0.745–2.049]
Grusi	3.03**	[1.234–7.448]
Gurma	0.94	[0.531–1.670]
Mande	2.80	[0.551–14.19]
Other	2.28	[0.570–9.111]
*Occupation*		
Not working	Ref	1,1
Working	1.45*	[1.015–2.060]
*Partner’s education*		
No formal education	Ref	1,1
Primary	0.98	[0.636–1.509]
Secondary+	1.12	[0.756–1.659]
*Health decision making capacity*		
Alone	Ref	1,1
Not alone	1.25	[0.901–1.722]
*Hold a valid NHIS card*		
No	Ref	1,1
Yes	1.24	[0.806–1.909]
*Getting medical help for self: Distance is a problem*		
Big problem	Ref	1,1
Not a big problem	1.63***	[1.239–2.145]

Sources: GDHS 2014, AOR = Adjusted Odds Ratio, CI=Confidence Interval in square brackets; Ref = Reference Category; *p < 0.05, **p < 0.01, ***p < 0.001

## Discussion

This study aimed at investigating the prevalence and correlates of PNC utilisation among women aged 15–49 in rural Ghana. In the multivariate regression model, four variables showed significant association with PNC and these are ecological zone, ethnicity, occupation and whether distance to health facility was problematic or otherwise. The direction of significance of these variables are discussed in this section of the manuscript. On ecological zone, we realised that women who resided in the Savanna zone were more probable to utilise PNC as compared to those in the Coastal zone. Comparatively, the Coastal zone is well resourced and endowed with more health facilities and health workforce compared to the Savanna zone [25]. However, this study plausibly strengthens the position of the socio-behavioural model that healthcare utilisation do not only depend on availability of health facilities or services but personal assessment of the need for the service, personal traits and beliefs [26, 27]. Ecological variation has similarly been observed as a determinant of PNC in Malawi [28]. Specifically, they noted that the odds of utilising PNC was 46% less among women in the central region and 53% less among women in the southern region than women in the northern region of Malawi [28].

Our study revealed that Guan women were more probable to utilise PNC than the Akan. Several studies have also found disparity in maternal healthcare utilisation across ethnic lines [29–32]. In explaining differentials in maternal healthcare utilisation across ethnicity, scholars argue that ethnicity has a link with social stratification in most contexts, and people who belong to ethnic minorities tend to be marginalised and discriminated against, and this adversely affect their prospects of utilising available services and opportunities [33]. However, our study failed to provide reasons why the Guan, being minority, were more inclined to PNC as compared to the Akan. Hence, further studies on ethnicity and utilisation of PNC in Ghana may be required.

We observed that women who were working were more probable to utilise PNC as compared to those not working. The results are in line with a Uganda based study by Ndugga, Namiyonga and Sebuwufu [34] who realised that unemployed women had lower odds of attending postnatal care compared with women who were working. Our results are also congruent with Malawian based study which noted that mothers who were working were 44% more likely to be checked by a professional health worker within 42 days of delivery than women who were jobless [28]. A plausible explanation is that unemployed women are likely to be less endowed economically and as a result may be dissuaded from utilising healthcare due to the associated cost even if such services are recommended by health workers [35, 36]. This may underscore the exigency to create employment avenues and opportunities for women in order to enhance their prospects of utilising PNC.

Finally, in agreement with a previous study [34], the present study revealed that women who viewed distance to health facility as unproblematic were more likely to utilise PNC as compared to those that perceived it as problematic. Malawi based study also indicated that women who perceived that distance to health facility was not a hindrance to their access to health care were more likely to attend early postnatal care than those who perceived distance to the facility as a problem [37]. Izudi et al. [38] also contended that long distances limit the willingness and ability of postpartum women to seek PNC due to the physical difficulties of travel and high cost of motorized transport. The finding highlights the need to reconsider the availability and citing of maternal healthcare services. During the postnatal period, women may be recovering from childbirth and they may not have enough strength to cover long distances [39]. As such, increasing maternal healthcare centers in rural communities and citing these facilities at shorter intervals within communities can substantially reduce the challenge posed by distance.

### Strengths and weaknesses

The study utilises data from a nationally representative survey and applies rigorous analytical procedures in estimations. Another key strength of the study is its focus on rural women, a group that have not received much attention as far as studies on PNC in Ghana is concerned. The limitations of the study include the cross sectional study design, which do not allow causal inference. There is also a possible recall bias on the part of the surveyed women.

## Conclusions

This study investigated factors associated with PNC among rural residents in Ghana. The study showed that ethnicity, ecological zone, occupation and distance to health facility predict PNC utilisation among rural residents of Ghana. The study has policy and practical implications on maternal healthcare provision in Ghana. First, the study points to the need for government to increase maternal healthcare facilities in rural settings in order to reduce the distance covered by women to access PNC. Second, enhancing empowerment and economic opportunities of women may be required to improve PNC utilisation among the rural populace of Ghana. Further study, preferably qualitative, may be needed to unveil the ethnic driven variation in PNC utilisation among rural women in Ghana.

## Supplementary Information

**Additional file 1.**

**Additional file 2.**

**Additional file 3.**

## Data Availability

The datasets generated and/or analysed during the current study are available in the Measure DHS repository, www.measuredhs.org.

## Abbreviations

AOR: Adjusted Odds Ratio
GDHS: Ghana Demographic and Health Survey
GSS: Ghana Statistical Service
GHS: Ghana Health Service
ICF: Inner-City Fund
NPHRL: National Public Health Reference Laboratory
NHIS: National Health Insurance Scheme
PNC: Postnatal Care
SDG: Sustainable Development Goal
SSA: Sub-Saharan Africa
VIF: Variance Inflation Factor
WHO: World Health Organisation
