# Supplementary material for: Postnatal care utilisation among women in rural Ghana: analysis of 2014 Ghana demographic and health survey
Source: BMC Pregnancy Childbirth. 2021 Jan 7;21:26. doi: 10.1186/s12884-020-03497-4 (PMC7791732; doi:10.1186/s12884-020-03497-4)
Supplement: Supplementary file 1 — Additional file 1. [file 12884_2020_3497_MOESM1_ESM.docx]

**Appendix 1: Linktest results**

| LIN | Coef. | Std Err | z | p>/z/ | 95% CI |
| --- | --- | --- | --- | --- | --- |
| _hat | 0.724 | 0.171 | 4.25 | 0.000 | [0.390-1.058] |
| _hatsq | 0.145 | 0..082 | 1.75 | 0.080 | [-0.017-0.306] |
| _cons | 0.023 | 0.097 | 0.24 | 0.810 | [-0.017-0.213] |
