# Supplementary material for: Postnatal care utilisation among women in rural Ghana: analysis of 2014 Ghana demographic and health survey
Source: BMC Pregnancy Childbirth. 2021 Jan 7;21:26. doi: 10.1186/s12884-020-03497-4 (PMC7791732; doi:10.1186/s12884-020-03497-4)
Supplement: Supplementary file 2 — Additional file 2. [file 12884_2020_3497_MOESM2_ESM.docx]

**Appendix 2: Logistic model of PNC Goodness-of-fit test**

| Number of observation | 1442 |
| --- | --- |
| Number of covariate patterns | 594 |
| Pearson Chi2(571) | 633.10 |
| Prob>chi2 | 0.0364 |
