# Supplementary material for: Postnatal care utilisation among women in rural Ghana: analysis of 2014 Ghana demographic and health survey
Source: BMC Pregnancy Childbirth. 2021 Jan 7;21:26. doi: 10.1186/s12884-020-03497-4 (PMC7791732; doi:10.1186/s12884-020-03497-4)
Supplement: Supplementary file 3 — Additional file 3. [file 12884_2020_3497_MOESM3_ESM.docx]

**Appendix 3: Multicollinearity Test**

| Variable | VIF | 1/VIF |
| --- | --- | --- |
| Ecological zone | 1.63 | 0.612323 |
| Partner’s education | 1.58 | 0.631613 |
| Wealth status | 1.36 | 0.735742 |
| Religion | 1.16 | 0.858727 |
| Marital status | 1.09 | 0.920219 |
| Hold a valid NHIS card | 1.08 | 0.922650 |
| Getting medical help: distance is a problem | 1.06 | 0.941437 |
| Ethnicity | 1.02 | 0.976997 |
| Health decision making | 1.02 | 0.980464 |
| Occupation | 1.02 | 0.981627 |
| Mean VIF | 1.20 | |
